# Cerebral Oxygen Saturation Associates with Changes in Oxygen Transport Parameters during Cardiopulmonary Bypass

**DOI:** 10.3390/jpm14070691

**Published:** 2024-06-27

**Authors:** Christoph Eisner, Heimo Adam, Markus A. Weigand, Aleksandar R. Zivkovic

**Affiliations:** 1 Department of Anesthesiology, Medical Faculty Heidelberg, Heidelberg University, 69120 Heidelberg, Germany; markus.weigand@med.uni-heidelberg.de; 2 Department of Cardiovascular Perfusion, Medical Faculty Heidelberg, Heidelberg University, 69120 Heidelberg, Germany; heimo.adam@med.uni-heidelberg.de

**Keywords:** near-infrared spectroscopy, NIRS, goal-directed perfusion, transesophageal echocardiography, TEE, DuBois, cardiac surgery

## Abstract

(1) Background: Adequate organ perfusion during cardiopulmonary bypass (CPB) requires accurate estimation and adjustment of flow rates which conventional methods may not always achieve. Perioperative monitoring of cerebral oxygen saturation (ScO_2_) may detect changes in oxygen transport. This study aims to compare estimated and measured perfusion flow rates and assess the capacity of ScO_2_ to detect subtle changes in oxygen transport during CPB. (2) Methods: This observational study included 50 patients scheduled for elective coronary artery bypass grafting (CABG) surgery, all of whom provided written informed consent. Perfusion flow rates were estimated using the DuBois formula and measured using echocardiography and a flow probe in the arterial line of the CPB system. ScO_2_ was continuously monitored, alongside intermittent measurements of oxygen delivery and extraction ratios. (3) Results: Significant discrepancies were found between estimated flow rates (5.2 [4.8–5.5] L/min) and those measured at the start of the surgery (4.6 [4.0–5.0] L/min). These discrepancies were flow rate-dependent, being more pronounced at lower perfusion rates and diminishing as rates increased. Furthermore, ScO_2_ showed a consistent correlation with both oxygen delivery (r = 0.48) and oxygen extraction ratio (r = 0.45). (4) Conclusions: This study highlights discrepancies between estimated and actual perfusion flow rates during CPB and underscores the value of ScO_2_ monitoring as a continuous, noninvasive tool for maintaining adequate organ perfusion, suggesting a need for improved, patient-tailored perfusion strategies.

## 1. Introduction

Monitoring critical parameters during cardiac surgery is paramount for ensuring patient safety and successful outcomes. Hemodynamic metrics are particularly vital for maintaining adequate organ perfusion throughout the perioperative period [1,2,3]. Cardiopulmonary bypass (CPB), a cornerstone of modern cardiac surgery, remains a necessary component of most operative procedures. It allows precise control over a patient’s physiological parameters, leading to distinct considerations in patient management. With the evolution of CPB techniques, the emphasis on individualized, goal-directed perfusion strategies has become increasingly important [4,5,6,7]. The concept of goal-directed perfusion, which stems primarily from critical care medicine, represents a relatively novel approach in the realm of CPB [8,9]. Goal-directed perfusion involves the implementation of an appropriate and personalized hemodynamic strategy for each patient [10,11]. However, precise determination of the required perfusion levels poses challenges owing to various confounding factors that may introduce biases into the calculation [12]. Protocols traditionally based on body surface area calculations remain the most commonly employed method for estimating the required perfusion flow. However, they may have some variability and limitations in accuracy [13].

Current perioperative monitoring techniques are essential for managing these challenges. Near-infrared spectroscopy (NIRS) is one such method providing noninvasive, continuous monitoring of cerebral perfusion by measuring cerebral oxygen saturation (ScO_2_). NIRS has gained recognition for its role in the early identification and prevention of major perioperative events [14]. Although not considered mandatory, NIRS-based monitoring of ScO_2_ has gradually gained acceptance in standard operating practice [15,16,17]. The beneficial role of NIRS monitoring has been previously reported, including the early identification and prevention of major perioperative events [18,19,20,21,22,23,24]. NIRS has also been increasingly used to monitor perioperative fluctuations in cerebral oxygen supply and demand [25,26]. However, research on this subject has mostly been restricted to limited comparisons of ScO_2_ to hemodynamic and clinical parameters. Much less is known about the correlation of NIRS-based ScO_2_ measurements with oxygen transport parameters, that is, major indicators of adequate organ perfusion during CPB.

Inadequate tissue perfusion, which often occurs subtly, can initially remain unnoticed under standard monitoring [24,27]. Current state-of-the-art monitoring techniques, including perioperative echocardiography and NIRS, among others, play an important role in minimizing such events [28,29,30]. Continuous optimization of the target perfusion flow during CPB in each individual patient remains a continuous challenge.

This study aimed to evaluate the accuracy of current protocols for estimating adequate perfusion flow rates during CPB and to assess the capacity of NIRS in detecting subtle changes in oxygen transport during the procedure.

## 2. Materials and Methods

### 2.1. Study Population

**Figure 1:**
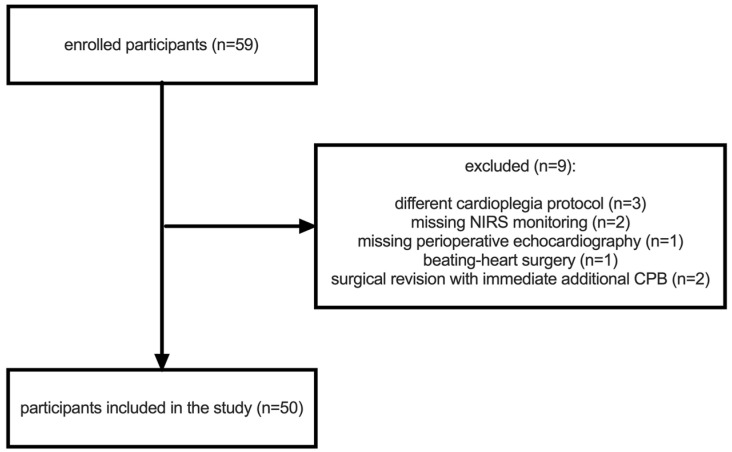
Flow chart showing the recruitment of the study population.

This observational clinical pilot study was approved by the Ethics Committee of the Medical Faculty of Heidelberg (Trial-Code no. S-246/2019) and was conducted in accordance with the principles of the Declaration of Helsinki and Good Clinical Practice. Written informed consent was obtained from the patients or their legal guardians. A total of 59 patients who were scheduled for elective coronary artery bypass grafting (CABG) surgery were screened for eligibility between May and July 2019. Inclusion criteria were: age ≥ 18 years, elective bypass surgery, and the capacity to provide informed consent. Exclusion criteria included: preoperative hemoglobin < 10 g/dL, hematocrit < 30%, preoperative end-stage renal disease (requiring renal replacement therapy), prior coronary artery bypass surgery, use of a cardioplegia solution other than Brettschneider, non-consent to data collection, expanded procedures including valve or aortic surgery, and off-pump coronary artery bypass (OPCAB) or “beating-heart” surgery. Nine patients were excluded from the study, three of whom were excluded due to receiving a different cardioplegia protocol: intermittent antegrade warm cardioplegia protocol (Figure 1).

### 2.2. Anesthesia

All patients received midazolam (7.5 mg) prior to surgery. Anesthesia was induced with sufentanil (0.5–1.0 µg/kg), propofol (1–2 mg/kg), or midazolam (0.1 mg/kg). Endotracheal intubation was facilitated using rocuronium (0.6 mg/kg). Anesthesia was maintained with sufentanil and sevoflurane (1–2.5%).

### 2.3. Extracorporeal Circulation

The extracorporeal perfusion circuit consisted of an S5^®^ (LivaNova, London, UK) heart–lung machine set in combination with either the Inspire 6F Dual^®^ (LivaNova, London, UK) oxygenator module with an integrated arterial filter and a hard-shell venous reservoir or the Affinity Fusion^®^ (Medtronic, Meerbusch, Germany) oxygenation system with an integrated venous reservoir. The priming solution consisted of 1000 mL of crystalloid solution (Sterofundin^®^ ISO, B. Braun, Melsungen, Germany) containing 10,000 IU of heparin. After achieving an activated clotting time (ACT) of greater than 450 s (typically requiring 400 IU/kg heparin), the aortic root was cannulated, followed by venous bicaval cannulation. A single dose of cold (5–8 °C) Bretschneider cardioplegia solution (Custodiol^®^, Dr. Franz Köhler Chemie GmbH, Bensheim, Germany) was administered following cross-clamping of the aorta. The administration was performed antegrade, with a volume of 2000 mL delivered via gravity through an aortic root cannula over a period of 6–7 min. The nonpulsatile pump flow was targeted at 2.5 L/min/m^2^. The target body temperature during perfusion was set at 34 °C. The body surface area was calculated using the DuBois formula:

Body Surface Area = 0.007184 × Height^0.725^ × Weight^0.425^ (1)

The perfusion flow rate was monitored using an ultrasound flow probe from the extracorporeal membrane oxygenation (ECMO) console (CentriMag^TM^ System, Abbott, Green Oaks, IL, USA). The probe was strategically placed in the arterial line of the CPB system, distal to all shunts, allowing for precise measurement of blood flow. This placement ensured an accurate assessment of the perfusion flow rate, considering the unique flow dynamics within the CPB circuit. The mean arterial pressure during the CPB was maintained with norepinephrine when necessary. CABG surgical procedures were performed according to hospital standards. After weaning from cardiopulmonary bypass, the heparin effect was antagonized by administering protamine sulfate (4 mg/kg).

### 2.4. Transesophageal Echocardiography (TEE)

Intraoperative TEE studies were performed by an attending anesthesiologist using a multiplane 4–7 MHz TEE probe and an iE33 ultrasound machine (Philips, Amsterdam, The Netherlands). The study included standard views and hemodynamic measurements as described in the current guidelines [31]. Echocardiographic determination of cardiac output was performed using the modified Bernoulli equation.

### 2.5. Near-Infrared Spectroscopy

Cerebral oxygen saturation was measured using an INVOS 5100C near-infrared spectroscopy monitor (Somanetics, Medtronic, Meerbusch, Germany). Changes in oxygenated hemoglobin were monitored by placing near-infrared light-transmitting optodes and light detectors on each fronto-temporal region, avoiding the frontal sinuses. Optodes were placed prior to anesthesia and remained positioned until the patient recovered from surgery. Cerebral oxygen saturation, expressed as a percentage, represents the ratio of oxygenated to total tissue hemoglobin.

### 2.6. Measurements

We defined three specific time points for measurements during the perioperative period: (1) surgery start—after anesthesia induction but prior to skin incision; (2) CPB on–commencement of the cardiopulmonary bypass; and (3) CPB off—termination of the cardiopulmonary bypass. At each time point, we recorded blood pressure, NIRS measurements (ScO_2_ for the left and right sides), and blood gas analysis (arterial and venous). Blood gas analysis included measurements of hemoglobin and lactate concentrations, as well as oxygen and carbon dioxide saturation and partial pressure. We calculated the values for oxygen delivery (DO_2_) and the oxygen extraction ratio using the corresponding formulas. Cardiac output was determined using TEE at the start of surgery. The pump flow rate of the heart–lung machine was recorded for the time points during cardiopulmonary bypass. Indexed parameters were normalized using body surface area, which was calculated using the DuBois formula.

### 2.7. Statistical Analysis

The study data were collected in an electronic database (Microsoft Excel for Microsoft 365 MSO, Version 2008, Microsoft Corp., Redmond, WA, USA). Statistical analyses were performed using GraphPad Prism 10 (GraphPad Software, La Jolla, CA, USA, www.graphpad.com, accessed on 3 April 2024). D’Agostino and Pearson omnibus normality tests were used to verify the Gaussian distribution of the study data. The results are presented as median values with interquartile ranges (IQRs). Statistical tests used in this study included repeated measures one-way ANOVA with Geisser–Greenhouse correction and Dunnett’s multiple comparisons test, mixed-effects analysis followed by Dunnett’s multiple comparisons test, the Friedman test followed by Dunn’s multiple comparison test for repeated measurements, and the Mann–Whitney test. Mixed-effects analysis was used to accommodate occasional random missing measurements, ensuring the analysis remained robust. Bland–Altman plots were used to compare the assay methods. Correlations were tested using Spearman’s correlation test. The best-fit value was calculated by using a simple linear regression model. Statistical significance was set at *p* < 0.05.

## 3. Results

This study included 50 patients who were scheduled for CABG surgery with cardiopulmonary bypass. Figure 1 and Table 1 present the recruited study population and the clinically relevant patient data.

### 3.1. Perioperative Hemodynamics

We initially analyzed the perioperative dynamics of several key hemodynamic and blood gas parameters of patients scheduled for CABG surgery. These parameters included the cardiac index, mean blood pressure, hemoglobin concentration, lactate concentration, and the partial pressures of arterial O_2_ (paO_2_) and CO_2_ (paCO_2_). Echocardiography revealed that the cardiac index, measured at the start of the procedure, was significantly lower than the required pump perfusion index both at the start and at the end of cardiopulmonary bypass (Figure S1a, Table S1). The initially measured mean blood pressure remained comparable throughout the procedure, with no significant differences observed at the start and at the end of cardiopulmonary bypass (Figure S1b, Table S1). The hemoglobin concentration significantly decreased upon initiating cardiopulmonary bypass and remained reduced during the procedure (Figure S1c, Table S1). The lactate concentration remained within standard laboratory values throughout the procedure. Despite a statistically significant increase at the end of cardiopulmonary bypass, the lactate level remained within the normal range (Figure S1d, Table S1). The partial pressure of arterial O_2_ and CO_2_ remained within the standard range throughout the procedure (Table S1). Additionally, mild hypothermia was maintained during the bypass (Table S1).

### 3.2. Flow Rate-Sensitive Discrepancy between Calculated and Measured Perfusion Flow Rates

**Figure 2:**
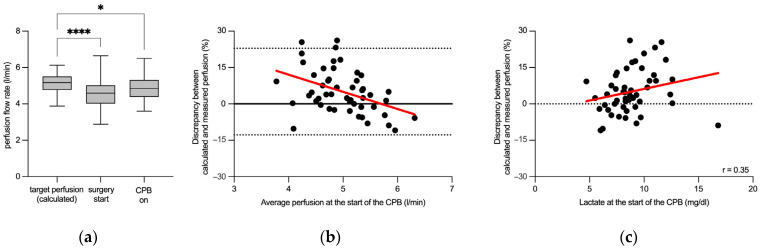
Differences between the calculated and measured perfusion flow rates. The box plots in panel (**a**) show the target perfusion flow rate (calculated before the procedure), cardiac output measured echocardiographically at the start of the surgery (surgery start), and the initial perfusion flow rate measured at the start of cardiopulmonary bypass (CPB on). Panel (**b**) features a Bland–Altman plot that highlights how the discrepancy between the calculated target and the measured perfusion flow rate at the start of CPB varies with changes in the pump flow rate, plotted against the average perfusion rate at this key time point. Panel (**c**) shows a correlation analysis between the discrepancy in calculated and measured perfusion flows and the lactate concentration at the start of CPB, underscoring the relationship between perfusion adequacy and metabolic response. In the box plots of panel (**a**), central lines signify median values, boxes indicate quartiles, and whiskers represent minimum and maximum values. The black dots in panels (**b**,**c**) represent individual data points, with the red line indicating the best-fit linear regression. The dotted lines in panel (**b**) depict the 95% limits of agreement. * *p* < 0.05; **** *p* < 0.0001 (Friedman test followed by Dunn’s multiple comparison test); r: Spearman’s correlation coefficient.

Next, we examined whether the targeted perfusion flow rate (calculated prior to surgery using the DuBois formula) corresponded to the echocardiographic measurements of cardiac output obtained after the start of surgery as well as the perfusion flow rate measured at the beginning of cardiopulmonary bypass. Indeed, a marked discrepancy was observed between the calculated target pump flow and two distinct measurements. The target pump flow (5.2 (4.8–5.5) L/min), calculated prior to surgery, differed significantly from both the echocardiographic measurement of cardiac output following the start of surgery (4.6 (4.0–5.0) L/min) and the measured perfusion flow after the initiation of cardiopulmonary bypass (4.9 (4.4–5.3) L/min; Figure 2a).

In addition, we investigated the relationship between the magnitude of the observed discrepancy and the rate of perfusion flow. We found that the quantitative discrepancy between the calculated and measured flows was more pronounced at lower perfusion flow rates and diminished as the flow rate increased, with the smallest difference (minimal discrepancy) observed at 5.7 L/min. Notably, at higher perfusion flow rates, a negative discrepancy was evident. This trend highlights the varying differences across the range of perfusion flows, as depicted in the Bland–Altman plot in Figure 2b. Nevertheless, the magnitude of the difference between the target and measured perfusion flow showed no correlation with the mean arterial pressure or hemoglobin concentration. However, the magnitude of the observed discrepancy correlated with the lactate concentration at the time of bypass initiation (Figure 2c).

### 3.3. Cerebral Oxygen Saturation Associates with Perioperative Changes in Oxygen Transport Parameters

We next examined whether cerebral oxygen saturation monitoring might help to better understand the observed discrepancies in perioperative perfusion flow assessment.

Bilateral cerebral oxygen saturation measurements (ScO_2_) were consistently taken and then averaged for each time point due to the absence of notable differences between the left and right ScO_2_ readings (Table S1). This approach ensured reliable data representation, reflecting initially stable cerebral oxygenation with a slight yet significant reduction in ScO_2_ toward the end of cardiopulmonary bypass. Despite this reduction, the median ScO_2_ values remained within clinically acceptable limits throughout the surgery (Figure S2a, Table S1). The correlation analysis revealed no significant associations between ScO_2_ and cardiac output/perfusion flow (Figure S2b) or between ScO_2_ and the cardiac/perfusion index. As anticipated, a notable correlation was identified between the ScO_2_ concentration and the hemoglobin concentration during surgery (Figure S2c). Further analysis of the relationship between ScO_2_ and paCO_2_ revealed a mild but significant correlation (Figure S2d).

**Figure 3:**
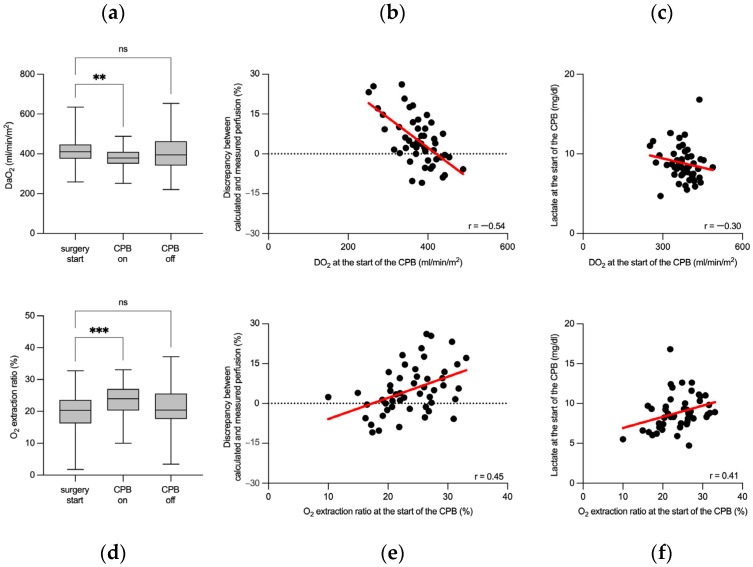
Perioperative oxygen transport fluctuations correlate with differences in flow rate sensitivity between calculated and measured perfusion rates. The box plots in panel (**a**) depict changes in oxygen delivery during cardiopulmonary bypass. Panel (**b**) plots the observed changes in the oxygen delivery at the start of cardiopulmonary bypass against the magnitude of the discrepancy between the calculated and actual measured perfusion flow rates at this time point. A scatter plot in panel (**c**) represents correlation analysis between oxygen delivery and the lactate concentration at the start of the cardiopulmonary bypass. The box plots in panel (**d**) show changes in the oxygen extraction ratio during cardiopulmonary bypass. Changes in the oxygen extraction ratio observed at the start of cardiopulmonary bypass are plotted against the magnitude of the discrepancy between the calculated and measured perfusion flow rates at this time point (**e**). Correlation analysis between oxygen extraction ratio and the lactate concentration at the start of the cardiopulmonary bypass is shown in panel (**f**). Central lines in the box plots indicate median values, boxes denote quartiles, and whiskers represent minimum and maximum values. Black dots represent individual measurements. The best-fit linear regression line is presented in red. DO_2_: oxygen delivery; CPB: cardiopulmonary bypass; ns: not significant; ** *p* < 0.01; *** *p* < 0.001 (repeated measures ANOVA followed by Dunnett’s multiple comparison test); r: Spearman’s correlation coefficient.

To further investigate the observed flow rate-sensitive perfusion discrepancy, we analyzed oxygen transport parameters during CPB. At the onset of CPB, DO_2_ indexed to body surface area significantly decreased from pre-CPB levels and returned to baseline by the end of CPB (Figure 3a, Table S1). This transient decrease in DO_2_ at the start of CPB was moderately correlated with the discrepancy between the estimated and actual measured perfusion flow rates, highlighting the importance of precise flow rate management during CPB (Figure 3b). The decrease in DO_2_ was further substantiated by a moderate negative correlation with lactate levels measured at the start of CPB (Figure 3c), underlining the metabolic response to changes in oxygen delivery. Similarly, the oxygen extraction ratio was examined, revealing an increase at the start of CPB, which normalized post-CPB (Figure 3d, Table S1). This initial increase in the oxygen extraction ratio was also moderately correlated with the discrepancy in the perfusion flow rate at CPB onset (Figure 3e), emphasizing the significance of accurate perfusion flow estimations for maintaining optimal oxygen transport. The association between elevated oxygen extraction ratios at the start of CPB and lactate concentrations confirmed the metabolic adjustments to the altered oxygen transport conditions during this critical phase (Figure 3f). Although the variations in oxygen transport parameters were statistically significant, all measurements remained within the clinically safe range.

**Figure 4:**
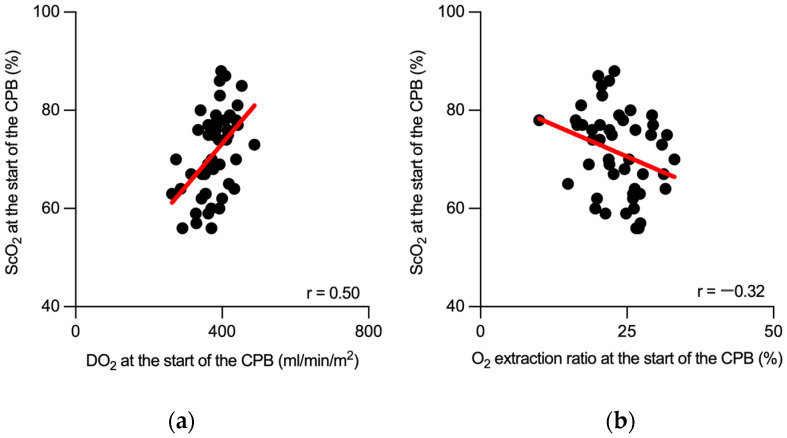
Correlations between cerebral oxygen saturation and oxygen transport parameters at the start of cardiopulmonary bypass. Scatter plots represent the correlation analysis between cerebral oxygen saturation and oxygen delivery (**a**) and the oxygen extraction ratio (**b**), measured at the start of cardiopulmonary bypass. Black dots represent individual measurements. The best-fit linear regression line is presented in red. ScO_2_: cerebral oxygen saturation; DO_2_: oxygen delivery; CPB: cardiopulmonary bypass; r: Spearman’s correlation coefficient.

Finally, we tested the potential role of perioperative ScO_2_ monitoring in detecting fluctuations in oxygen delivery during cardiopulmonary bypass. Initially, we focused on the correlation between ScO_2_ and DO_2_ at the start of CPB, revealing a stronger correlation that suggested a heightened sensitivity of ScO_2_ to initial perfusion changes (Figure 4a). This analysis was extended to encompass the entire surgical procedure, where the correlation, although significant, was less pronounced (Figure S3a). A comparable analysis was conducted for the oxygen extraction ratio, revealing similar patterns. ScO_2_ and the oxygen extraction ratio showed a distinct correlation in the initial phase of the CPB (Figure 4b). This correlation persisted throughout the procedure but was more subtle than that in the initial phase (Figure S3b). These findings highlight the value of ScO_2_ monitoring in identifying critical shifts in oxygen transport, particularly in the early stages of CPB.

## 4. Discussion

The current study reports a notable offset between the targeted pump perfusion rate of 2.5 L/min/m^2^ and the actual perfusion rate required during the procedure. Importantly, the extent of the observed discrepancy changes with the pump flow rate and correlates with lactate concentration. Furthermore, the magnitude of the discrepancy between calculated (using the DuBois method) and measured pump flow correlates with perioperative oxygen transport parameters in patients undergoing cardiopulmonary bypass. The second major finding was that continuous perioperative measurements of cerebral oxygen saturation correlate well with oxygen transport parameters, suggesting that this method is a sensitive, noninvasive, and simple tool for complementary perioperative monitoring of subtle changes in organ perfusion during cardiopulmonary bypass.

Adequate estimation of the perfusion required during cardiopulmonary bypass has been continuously optimized [13,14,32,33,34,35]. Nevertheless, a precise calculation remains a challenge for the accurate estimation of the required initial perfusion flow following the commencement of cardiopulmonary bypass.

Unexpectedly, our findings suggest that the DuBois-based calculation of the required perfusion flow may lead to underestimation, particularly in patients with lower body surface area. This indicates that the protocol may be more accurate when applied to patients with a greater body surface area or greater cardiac output. These findings contrast with the previously published work of Livingston and Lee [36]. These conflicting results can be partly explained by the nature of the calculations. However, we cannot exclude the possibility that the observed differences may be due to the variety of patient characteristics.

Furthermore, the lactate concentration, a reliable indicator of adequate organ perfusion, shows a modest correlation with the observed magnitude of the discrepancy between the targeted and measured pump flow rates, further validating the significance of our findings. This use of the lactate concentration aligns with broader practices in cardiac surgery and critical care medicine, where both the lactate and hemoglobin concentrations are recognized as established and reliable biomarkers for tissue ischemia [1,37,38,39,40,41,42,43].

Hemodilution, inherent to extracorporeal circulation, invariably leads to reduced hematocrit, hemoglobin concentration, and consequently oxygen delivery [5]. Although the oxygen extraction ratio is a well-established parameter for assessing oxygen transport and differentiating between flow-dependent and flow-independent oxygenation, its substantial fluctuations during the procedure are noteworthy. A key finding of this study is the correlation between cerebral oxygen saturation and the oxygen extraction ratio, demonstrating the capacity of ScO_2_ monitoring to reflect subtle changes in tissue perfusion. This highlights the potential of using ScO_2_ as a noninvasive, straightforward, and continuous technique that is critical for detecting subtle changes in organ perfusion during cardiopulmonary bypass. 

This study has several limitations. First, we cannot exclude the possibility that the observed ScO_2_ values are exclusively due to changes in CO_2_ concentration in the blood. Nevertheless, our data show rather stable CO_2_ values independent of the observed changes in ScO_2_, suggesting that this option is rather improbable. The small sample size and monocentric nature of the pilot study limit the ability to generalize the results. Furthermore, the study design included only patients scheduled for CABG. With a rather small sample size of adult patients, caution must be applied, as the findings might not be applicable to patients undergoing other cardiac surgeries or to the pediatric population. Next, the patients included in the study might have had a history of conditions and medications that could affect the interpretability of the data. The echocardiographic assessment of patient hemodynamics is admittedly operator-dependent to a certain extent. Finally, interpreting the absolute values of cerebral oxygenation obtained using near-infrared spectroscopy remains challenging owing to the technical limitations of the procedure. The scattering and attenuation effects of the light signal render the computation of the absolute values nearly impossible. Therefore, the advantage of measuring relative changes in regional cerebral oxygenation in a sensitive and noninvasive manner should be interpreted in conjunction with conventional monitoring. The comprehensive implementation of diverse monitoring procedures, including extensive interpretations thereof, is of utmost importance for optimal perfusion during cardiopulmonary bypass.

## 5. Conclusions

This study revealed that standard perfusion flow rate calculations might not be universally applicable. The observed discrepancies between targeted and actual perfusion flows vary with the perfusion rate, a phenomenon further substantiated by correlations with lactate concentrations and oxygen transport parameters. Additionally, the correlation between cerebral oxygen saturation and oxygen transport parameters emphasizes the ability of ScO_2_ monitoring to capture subtle shifts in organ perfusion during cardiopulmonary bypass. These findings suggest a need for nuanced perfusion strategies and underscore the importance of ScO_2_ monitoring in detecting subtle changes in organ perfusion.

## Figures and Tables

**Table 1:** Baseline characteristics and parameters of patients included in the study.

Number of Patients	50
Female/male (%)	7 (14%)/43 (86%)
Age (years)	66 (61−75)
BSA (m^2^)	2.1 (1.9−2.2)
NYHA I (%)	7 (14%)
NYHA II (%)	20 (40%)
NYHA III (%)	19 (38%)
NYHA not classified (%)	4 (8%)
History of single-vessel CAD	1 (2%)
History of double-vessel CAD	6 (12%)
History of triple-vessel CAD	43 (86%)
History of hypertension	47 (94%)
History of diabetes mellitus	13 (26%)
History of carotid artery stenosis	4 (8%)
ACC duration (minutes)	57 (47−77)
CPB duration (minutes)	102 (83−121)

ACC, aortic cross-clamp; BSA, body surface area; CAD, coronary artery disease; CPB, cardiopulmonary bypass; values are presented as median with quartiles unless otherwise noted.

## Data Availability

The data presented in this study are available on request from the corresponding authors.

## Supplementary Materials

The following supporting information can be downloaded at: https://www.mdpi.com/article/10.3390/jpm14070691/s1, Figure S1: Hemodynamic parameters during CABG surgery; Figure S2: Continuous measurement of cerebral oxygen saturation (ScO_2_) during CABG surgery; Figure S3: Correlations between cerebral oxygen saturation and oxygen transport parameters throughout the surgery; Table S1: Summary of the study results.
