# Supplementary material for: Cerebral Oxygen Saturation Associates with Changes in Oxygen Transport Parameters during Cardiopulmonary Bypass
Source: J Pers Med. 2024 Jun 27;14(7):691. doi: 10.3390/jpm14070691 (PMC11277785; doi:10.3390/jpm14070691)
Supplement: Supplementary file 1 [file jpm-14-00691-s001.zip › New Supplementary Figure 1.pdf]

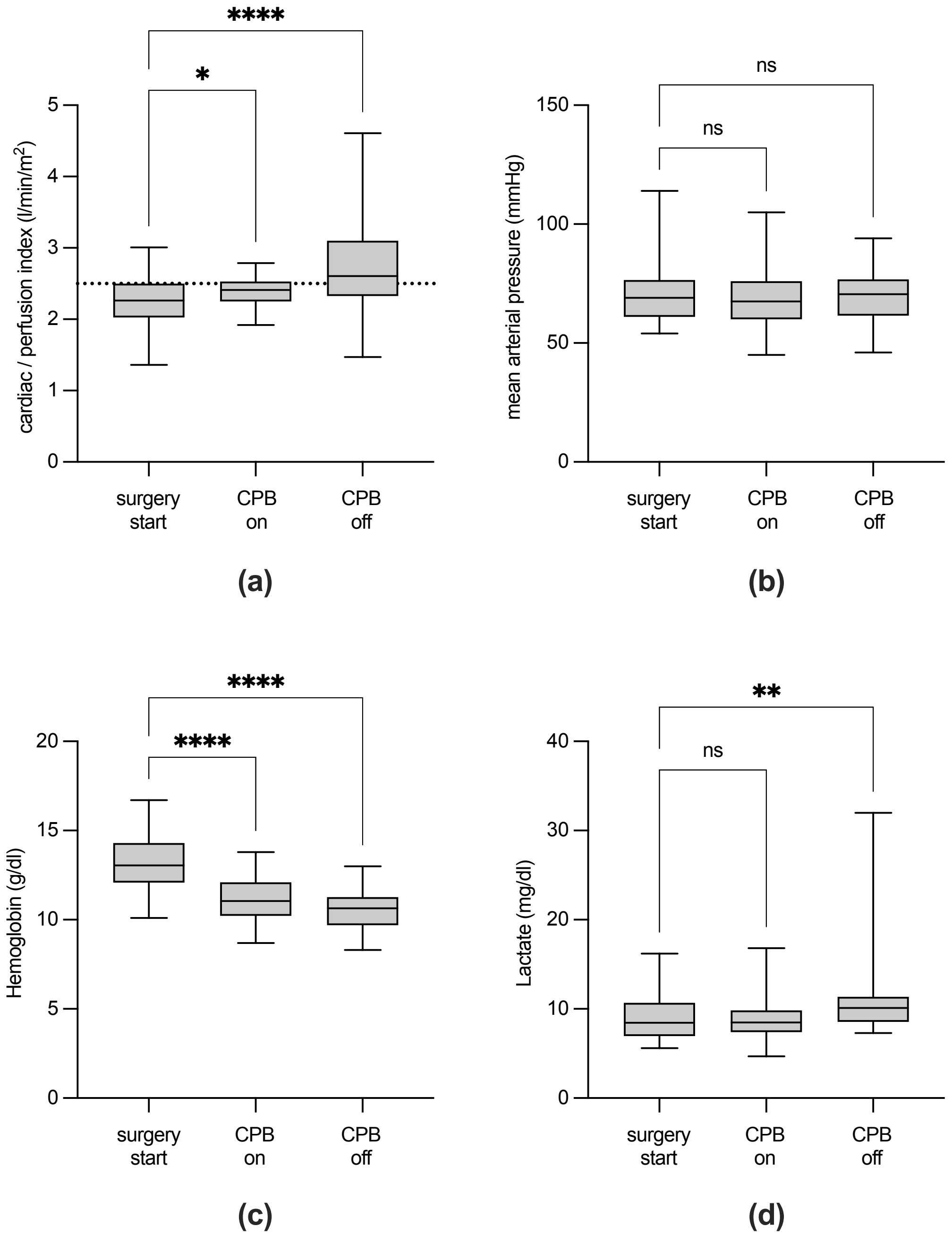

Figure S1: Hemodynamic parameters during CABG surgery. Panel (a) presents the cardiac index obtained via echocardiography prior to CPB (surgery start) and the perfusion pump flow rates measured at the initiation (CPB on) and conclusion (CPB off) of cardiopulmonary bypass. The dotted line indicates the calculated target value of 2.5 L/min/m2. Panel (b) depicts the mean arterial pressure measured perioperatively. Panels (c) and (d) present hemoglobin and lactate concentrations, respectively, obtained concurrently with the aforementioned measurements at key time points. Box plots display the median (central lines), interquartile range (boxes), and minimum and maximum values (whiskers). ns: not significant; * $p < 0.05$; ** $p < 0.01$; **** $p < 0.0001$ (repeated measures one-way ANOVA or mixed-effects analysis followed by Dunnett’s multiple comparison test); CPB: cardiopulmonary bypass.
