# Supplementary material for: Cerebral Oxygen Saturation Associates with Changes in Oxygen Transport Parameters during Cardiopulmonary Bypass
Source: J Pers Med. 2024 Jun 27;14(7):691. doi: 10.3390/jpm14070691 (PMC11277785; doi:10.3390/jpm14070691)
Supplement: Supplementary file 1 [file jpm-14-00691-s001.zip › New Supplementary Figure 2.pdf]

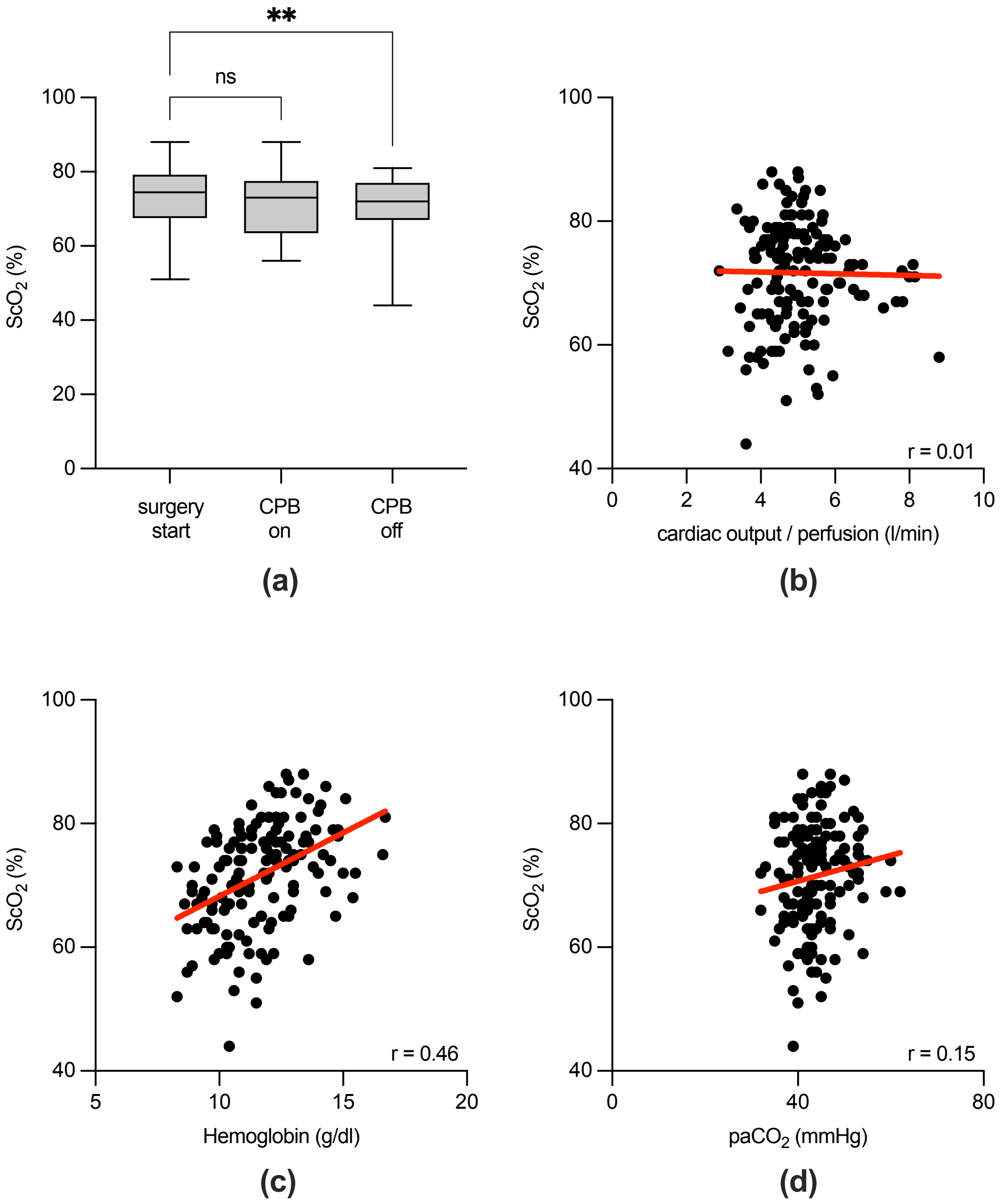

Figure S2: Continuous measurement of cerebral oxygen saturation (ScO_2) during CABG surgery. The violin plots in panel (a) represent the means of simultaneous bilateral ScO_2 measurements obtained by using fronto-temporal continuous near-infrared spectroscopy, recorded at the start of the surgery (surgery start), at the start of cardiopulmonary bypass (CPB on) and at the end of the cardiopulmonary bypass (CPB off). Scatter plots showing the correlation between ScO_2 and cardiac output/perfusion flow rate (b), hemoglobin concentration (c) and partial pressure of arterial carbon dioxide (d) measured throughout the procedure. Central lines in the box plots of panel (a) indicate median values, boxes denote quartiles and whiskers represent minimum and maximum values. Black dots in panels (b), (c) and (d) represent individual data points, with the red line indicating the best-fit linear regression. ns: not significant; ** $p < 0.01$ (mixed-effects analysis followed by Dunnett's multiple comparison test); r: Spearman's correlation coefficient; $paCO_2$: partial pressure of arterial carbon dioxide.
