# Supplementary material for: Cerebral Oxygen Saturation Associates with Changes in Oxygen Transport Parameters during Cardiopulmonary Bypass
Source: J Pers Med. 2024 Jun 27;14(7):691. doi: 10.3390/jpm14070691 (PMC11277785; doi:10.3390/jpm14070691)
Supplement: Supplementary file 1 [file jpm-14-00691-s001.zip › Supplementary Figure 3.pdf]

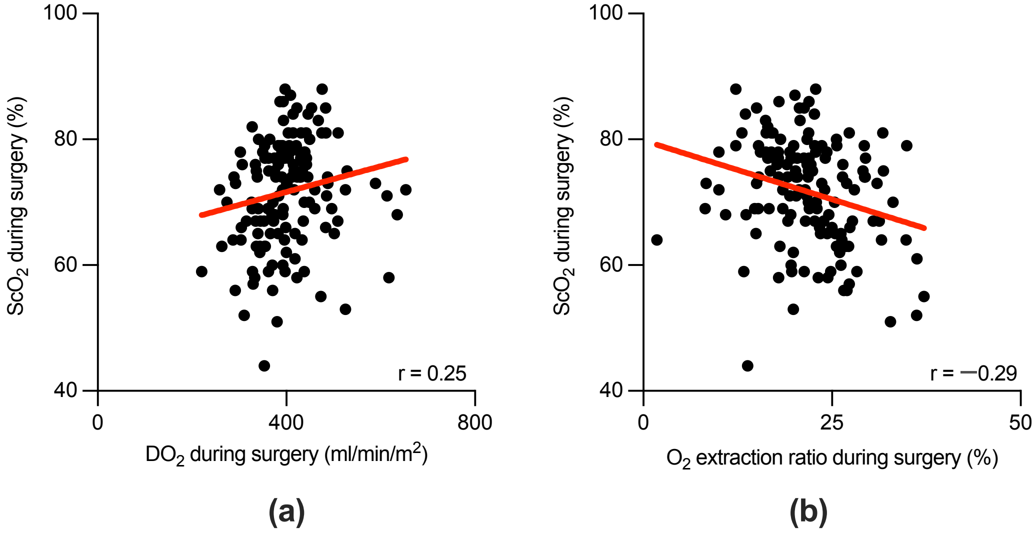

Figure S3: Correlations between cerebral oxygen saturation and oxygen transport parameters throughout the surgery. Panels represent the correlation analysis between cerebral oxygen saturation and oxygen delivery (a) and the oxygen extraction ratio (b), obtained through the surgery. Black dots represent individual measurements. The best-fit linear regression line is presented in red. ScO_2: cerebral oxygen saturation; DO_2: oxygen delivery; CPB: cardiopulmonary bypass; r: Spearman's correlation coefficient.
