# Supplementary material for: Cerebral Oxygen Saturation Associates with Changes in Oxygen Transport Parameters during Cardiopulmonary Bypass
Source: J Pers Med. 2024 Jun 27;14(7):691. doi: 10.3390/jpm14070691 (PMC11277785; doi:10.3390/jpm14070691)
Supplement: Supplementary file 1 [file jpm-14-00691-s001.zip › Supplementary Table 1.pdf]

Supplementary Table 1. Summary of the study results

parameter	surgery start	CPB on	CPB off
CO/perfusion (l/min)	4.6 (4.0 – 5.0)	4.9 (4.4 – 5.3)	5.5 (4.7 – 6.4)
CI/perfusion index ($l/min/m^2$)	2.3 (2.0 – 2.5)	2.4 (2.3 – 2.5)	2.6 (2.3 – 3.1)
MAP (mmHg)	69 (61 – 77)	68 (60 – 76)	71 (62 – 77)
Body temperature (°C)	36.2 (35.8 – 36.5)	35.7 (35.2 – 36.0)	36.7 (36.4 – 37.0)
Hemoglobin (g/dl)	13 (12 – 14)	11 (10 – 12)	11 (10 – 11)
Lactate (mg/dl)	8.5 (7.0 – 11.0)	8.5 (7.4 – 9.9)	10.0 (8.6 – 11.0)
$paCO_2$ (mmHg)	45 (41 – 49)	44 (43 – 50)	42 (39 – 45)
paO_2 (mmHg)	209 (110 – 314)	246 (191 – 308)	181 (119 – 278)
DaO_2 ($ml/min/m^2$)	410 (376 – 447)	379 (351 – 409)	394 (341 – 463)
O_2 extraction rate (%)	20 (16 – 24)	24 (20 – 27)	20 (18 – 26)
ScO_2 left (%)	74 (67 – 80)	71 (64 – 77)	71 (66 – 76)
ScO_2 right (%)	74 (68 – 80)	72 (65 – 78)	72 (64 – 75)
ScO_2 average (%)	75 (68 – 79)	73 (64 – 78)	72 /67 – 77)

CPB: cardiopulmonary bypass; MAP: mean arterial pressure; CO: cardiac output; CI: cardiac index; DaO_2: oxygen delivery; ScO_2: cerebral oxygen saturation; paO_2: arterial oxygen partial pressure; $paCO_2$: arterial CO_2 partial pressure; values represent medians with quartiles.
